# Supplementary figures and images for: Impaired motor-to-sensory transformation mediates auditory hallucinations
Source: PLoS Biol. 2024 Oct 3;22(10):e3002836. doi: 10.1371/journal.pbio.3002836 (PMC11449488; doi:10.1371/journal.pbio.3002836)

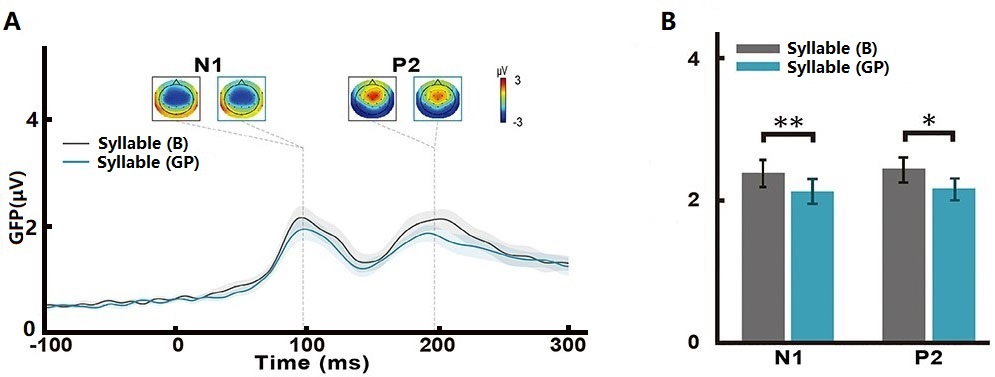

Supplement: S1 Fig — (A) ERP time course and topographic responses for GP and B conditions in normal populations. (B) Mean GFP amplitude at N1 and P2 latencies for GP (blue) and B (gray) conditions in normal populations (adapted from [39]). In the normal population, the amplitude of early N1 response in GP was less than that in B (t(18) = 3.406, p = 0.003, d = 0.3). The amplitude of the later P2 response in GP was reduced relative to B (t(18) = 2.240, p = 0.038, d = 0.342). These results suggested that CD that was induced during GP suppressed auditory responses. (This Figure reproduced with permission from the original publisher [39] and Oxford University Press (license number 5858081359804).) The underlying data for this figure can be found at http://osf.io/rsnu4/ and in S2 Data. (JPEG) [file pbio.3002836.s001.jpeg]

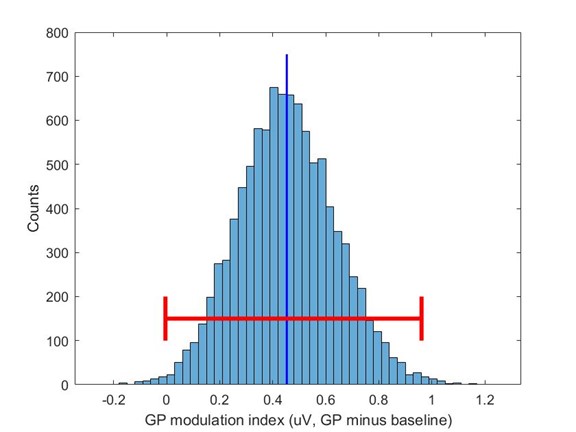

Supplement: S2 Fig — Bootstrapping results of N1 component in GP condition of AVH group. The simulation is done by resampling with replacement 10,000 times in the GP modulation index of the empirical sample (GP minus baseline). The blue vertical line denotes the mean of the empirical sample. The horizontal red bar indicates the 99% confidence interval (CI). The suppression (modulation index value less than 0) is outside the CI. The underlying data for this figure can be found at http://osf.io/rsnu4/ and in S2 Data. (JPG) [file pbio.3002836.s002.jpg]

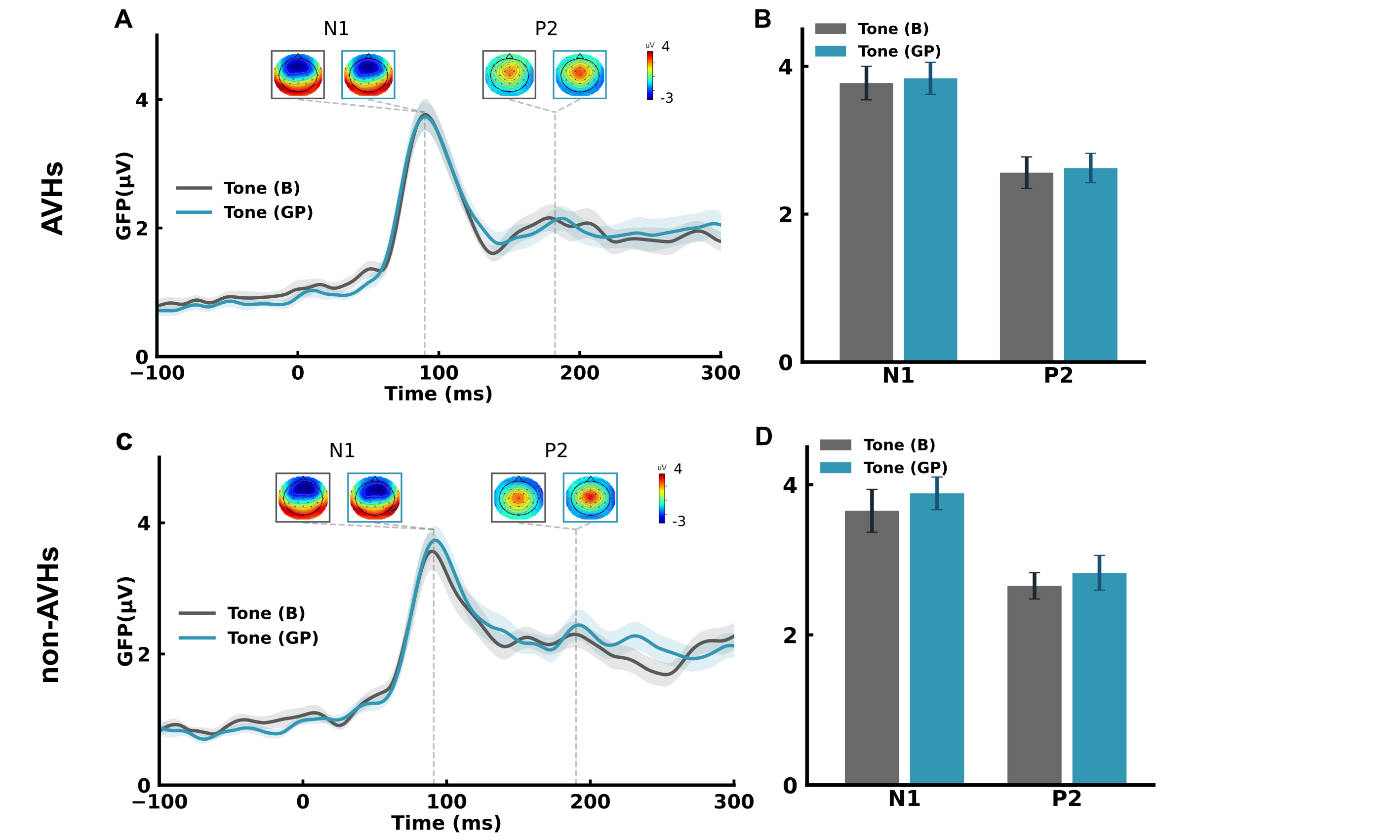

Supplement: S3 Fig — (A) ERP time course and topographic responses for GP and B conditions in AVHs patients. Typical N1 and P2 components were observed in the GFP waveforms for each condition. The response topographies at each peak latency are shown in boxes with the same color code of conditions. (B) Mean GFP amplitude at N1 and P2 latencies for GP (blue) and B (gray) conditions in AVHs patients. (C) ERP time course and topographic responses for GP and B conditions in non-AVHs patients. (D) Mean GFP amplitudes at the N1 and P2 latencies for GP (blue) and B (gray) conditions in non-AVHs patients. No significant differences between GP and B were observed in either group. Error bars indicate ± SEMs. The underlying data for this figure can be found at http://osf.io/rsnu4/ and in S2 Data. (TIF) [file pbio.3002836.s003.tif]

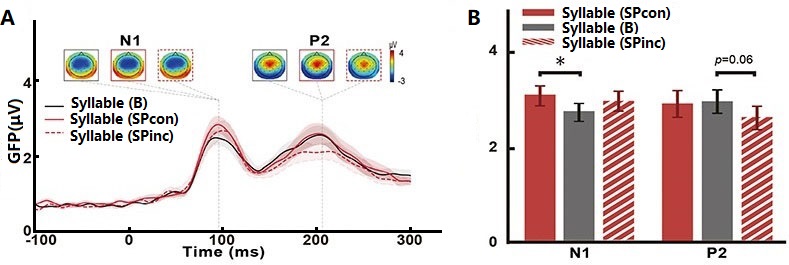

Supplement: S4 Fig — (A) ERP time course and topographic responses for SP and B conditions in non-AVHs patients. (B) Mean GFP amplitudes at N1 and P2 latencies for SP and B conditions. Responses in SPcon were significantly larger than those in B in N1 components (adapted from [39]). In the SP, early neural responses of N1 were larger than that in B when the auditory syllables were congruent with the SP visual cues (SPcon) (t(15) = −2.49, p = 0.025, d = −0.432). The effect was not significant in the later auditory responses of P2 (t(15) = 0.248, p = 0.808, d = 0.039). However, when the auditory syllables were incongruent with the specific preparation (SPinc), the effect in N1was not significant (t(15) = −1.48, p = 0.160, d = −0.283), nor in P2 (t(15) = 2.024, p = 0.061, d = 0.342). These results suggested that motor signals during SP modulated the perceptual responses based on the content congruency. (This figure reproduced with permission from the original publisher [39] and Oxford University Press (license number 5858081359804).) The underlying data for this figure can be found at http://osf.io/rsnu4/ and in S2 Data. (JPEG) [file pbio.3002836.s004.jpeg]

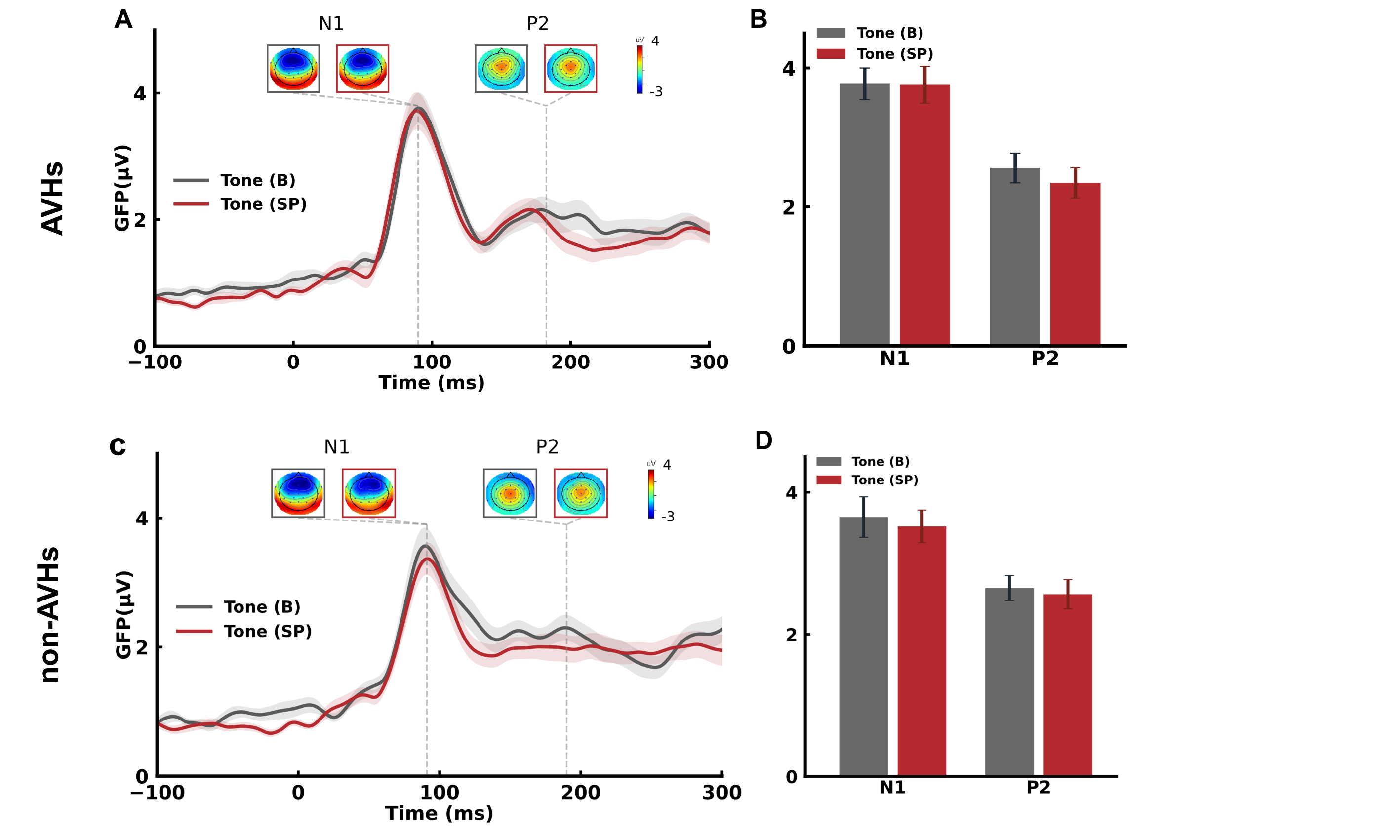

Supplement: S5 Fig — (A) ERP time course and topographic responses for SP and B conditions in AVHs patients. Typical N1 and P2 components were observed in the GFP waveforms of each condition. The response topographies at each peak latency are shown in boxes with the same color code of conditions. (B) Mean GFP amplitude at N1 and P2 latencies for SP (red) and B (gray) conditions in AVHs patients. (C) ERP time course and topographic responses for SP and B conditions in non-AVHs patients. (D) Mean GFP amplitudes at the N1 and P2 latencies for SP (red) and B (gray) conditions in non-AVHs patients. No significant differences between SP and B were observed in either group. Error bars indicate ± SEMs. The underlying data for this figure can be found at http://osf.io/rsnu4/ and in S2 Data. (TIF) [file pbio.3002836.s005.tif]
