# Supplementary material for: Impaired motor-to-sensory transformation mediates auditory hallucinations
Source: PLoS Biol. 2024 Oct 3;22(10):e3002836. doi: 10.1371/journal.pbio.3002836 (PMC11449488; doi:10.1371/journal.pbio.3002836)
Supplement: S1 Table — The underlying data for this figure can be found at http://osf.io/rsnu4/ and in S3 Data. (DOCX) [file pbio.3002836.s006.docx]

### Table S1. Demographics of AVHs, non-AVHs, and Normal Controls

|  |  |  |  |  |  |  |  |  |  |
| --- | --- | --- | --- | --- | --- | --- | --- | --- | --- |
|  | AVHs  (n=20) | non-AVHs (n=20) | Normal Controls for GP (n=19) | F or χ^2^ | *p* | Normal Controls for SP (n=16) | F or χ^2^ | *p* |  |
| Gender (M/F) | 10/10 | 14/6 | 5/14 | 7.45 | 0.024 | 5/11 | 5.39 | 0.07 |  |
| Age (years) | 25.60 (6.75) | 30.75 (6.15) | 23.89 (2.96) | 8.06 | 0.001 | 23.13 (3.38) | 8.40 | 0.001 |  |
| Education (years) | 13.00 (2.27) | 14.08 (3.16) | 16.53 (2.50) | 8.87 | 0.001 | 15.81 (2.79) | 4.64 | 0.014 |  |
| Age of onset | 20.85 (6.51) | 25.20 (7.70) | N/A | 1.93 | 0.06 | N/A | N/A | N/A |  |
| Duration (month) | 68.50 (65.22) | 66.00 (46.27) | N/A | 0.14 | 0.89 | N/A | N/A | N/A |  |
| PANSS total score | 76.50 (15.58) | 67.75 (13.91) | N/A | 1.87 | 0.07 | N/A | N/A | N/A |  |
| P3 subscore | 4.35 (1.27) | 1.00 (0.0) | N/A | 11.82 | <0.001 | N/A | N/A | N/A |  |
| P subscore | 21.25 (5.39) | 17.10 (4.06) | N/A | 2.75 | 0.009 | N/A | N/A | N/A |  |
| N subscore | 18.50 (4.47) | 17.90 (4.48) | N/A | 0.42 | 0.67 | N/A | N/A | N/A |  |
| G subscore | 36.75 (8.78) | 32.75 (8.08) | N/A | 1.50 | 0.14 | N/A | N/A | N/A |  |
| AVHs total score | 26.15 (4.42) | N/A | N/A | N/A | N/A | N/A | N/A | N/A |  |
