# Supplementary material for: Impaired motor-to-sensory transformation mediates auditory hallucinations
Source: PLoS Biol. 2024 Oct 3;22(10):e3002836. doi: 10.1371/journal.pbio.3002836 (PMC11449488; doi:10.1371/journal.pbio.3002836)
Supplement: Attachment — Submitted filename: PLOSBIO_Response to reviewers.docx [file pbio.3002836.s011.docx]

Dear Editor Dr. Schnell,

We thank you and the reviewers for your constructive comments and appreciation of the significance and novelty of our study. According to the detailed suggestions, we have thoroughly revised our manuscript. The major updates include

1) performed a bootstrap resampling analysis; the obtained confidence interval suggested that the predicted absence of suppression in the AVH group was unlikely due to chance

2) carried out correlation analyses between the severity of hallucination symptoms and neural measures; the significant positive correlation between the symptoms and deficits in neural indices, complementary to the predicted null effects, provided strong evidence supporting the proposed hypothesis

3) added details about the classification and evaluation of patient subgroups, elucidated EEG and modeling methodological procedures, as well as provided clarifications throughout the manuscript.

We believe the extensive additional analyses and new results significantly boost the conclusiveness of the study and hence our manuscript has been significantly improved. Please find our point-by-point responses below each comment.

Yours sincerely,

Xing Tian

(on behalf of Fuyin Yang and other authors)

Reviewer: #1

*In this manuscript, Li & Colleagues have examined an interesting aspect of corollary discharge (CD) / efference copy (EC) concept in terms of the seemingly counterintuitive & competing functions of action-induced suppression versus action-induced enhancement - in terms of its applicability to understand to the pathogenetic basis of auditory hallucinations in schizophrenia (SZ). To this effect, they examined SZ patients with & without auditory verbal hallucinations (AVH) in comparison with healthy controls. This study uses the experimental paradigm that differentiated CD versus EC. It is critical that the authors clarify the following queries to facilitate interpretations of their study findings.*

We thank you for the constructive comments. According to your insightful suggestions, we have conducted symptom-neural correlation analyses and provided clarifications throughout the manuscript. Please see our responses to each comment below.

*1) Did they find any relationship between negative symptom score and CD/EC metrics*

Re: Thank you for mentioning this important aspect of exploration. After testing the data for normality, we performed a Pearson correlation analysis to investigate the relationship between the neural responses and the scores of clinical symptoms. For the negative symptoms, we did not find any significant correlations between the negative symptom scores and CD/EC metrics in AVHs or non-AVHs groups. We have added a statement of these null results in the results section and copied below.

[in Results:]

‘We further explored the relations between neural measures of CD/EC and negative symptoms. Pearson correlation analyses did not reveal any significant correlation between negative symptom scores and CD/EC metrics in AVHs or non-AVHs groups.’

*2) I am curious to understand whether there was any relationship between noisy EC and AVH severity*

Re: We thank you for emphasizing this crucial aspect to test our hypothesis at a finer individual level. According to our hypothesis, the positive symptoms of auditory hallucinations ― random perceptual-like experiences without corresponding acoustic stimulations ― would be mediated by noisy EC that could activate multiple neighboring auditory neural representations around the sensory target of the action. That is, the noisy EC modulates and enhances the sensitivity of neural responses to unprepared auditory units. To test this hypothesis regarding EC and AVH, a difference score was calculated by subtracting the N1 response amplitude in the B condition from the SPinc condition in the AVHs group, representing the magnitude of the N1 enhancement effect in the SPinc condition. We found that the N1 enhancement magnitude significantly and positively correlated with the AHRS total scores (r = 0.562, p = 0.01; added as the new Fig. 5B in the manuscript and copied below as Fig. R1). That is, the more severe the AVHS symptoms, the more enhancement in the N1 responses in the SPinc condition. The results suggest that the significant enhancement of N1 response magnitude in SPinc condition in the AVH group was related to the degree of AVH symptom severity. This noisy and imprecise EC in AVHs may relate to the ‘non-sense’ and various forms of auditory hallucinations. We have added the new results as a new subsection in the Results section and copied below.

*Figure R1.* **The correlation between AHRS total scores and SPinc modulation index in AVHs group.** AHRS (Auditory Hallucinations Rating Scale) total scores represent the severity of auditory hallucinations symptoms; SPinc modulation index represents the magnitude of the N1 enhancement effects in the SPinc condition.

[in Results:]

**‘**According to our hypothesis about EC, the positive symptoms of auditory hallucinations ― random perceptual-like experiences without corresponding acoustic stimulations ― would be mediated by noisy EC that could activate neighboring auditory neural representations around the sensory target of the action. That is, the noisy EC modulates and enhances the sensitivity of neural responses to unprepared auditory units. To test this hypothesis, a difference score was first calculated by subtracting the N1 response amplitude in the B condition from the SPinc condition in the AVHs group. The difference score represents the magnitude of the N1 enhancement effects in the SPinc condition. The N1 enhancement magnitude significantly and positively correlated with the AHRS total scores (Fig.5B, *r* = 0.562, *p* = 0.01 ). That is, the more severe the AVHS symptoms, the more enhancement in the N1 responses in the SPinc condition. These results suggest that the enhancement of N1 response magnitude to the unprepared syllables was related to the degree of AVH symptom severity ― noisy and imprecise EC in AVHs may relate to the various forms of auditory hallucinations.’

[in Discussion:]

‘In AVH patients, the motor signals in specific preparation enhanced the neural responses to the unprepared syllables (Fig. 4). And the modulation effects of enhancement were positively correlated with the severity of AVH symptoms (Fig. 5B).’

*3) Please clarify whether PANSS was rescaled*

Re: We did not rescale the PANSS scores in this study. Rescaled PANSS is mainly applicable when evaluating changes in symptom severity, such as comparing and tracking changes over time (e.g. Leucht et al. 2007&2009). In our study, we implemented a cross-sectional assessment at one time of individual enrollment. As long as the assessment is consistent across participants, whether ‘0’ or ‘1’ representing ‘no symptom’ would not change the assessment of symptom severity at the time of participants’ enrollment. Moreover, we performed all the analyses with the rescaled PANSS data to verify and we found similar results to the ones obtained with the original PANSS scores. We have added clarifications in the Methods section.

Reference:

Leucht S, Davis JM, Engel RR, Kane JM, Wagenpfeil S. Defining ‘response’ in antipsychotic drug trials: recommendations for the use of scale-derived cutoffs. Neuropsychopharmacology. 2007;32:1903–1910.

Leucht S, Davis JM, Engel RR, Kissling W, Kane JM. Definitions of response and remission in schizophrenia: recommendations for their use and their presentation. Acta Psychiatr Scand. 2009;119(suppl. 438):5–12.

[in Methods:]

‘Trained psychiatrists implemented a one-time cross-sectional psychotic symptoms assessment at individual enrollment using the Positive and Negative Syndrome Scale (PANSS). The PANSS includes 30 items that measure the presence and severity of positive, negative, and general symptoms on a seven-point scale ranging from 1 to 7.’

*4)* *Did AVH & non-AVH groups differ in medication profile*

Re: In our study, all enrolled patients were treated with one or two second-generation oral antipsychotic drugs (e.g. olanzapine, risperidone, aripiprazole, clozapine, quetiapine, amisulpride). We chose olanzapine as the standard drug, for every patient, the cumulative antipsychotic dose was transformed into the equivalent dose to olanzapine based on defined daily doses (DDDs) presented by the World Health Organisation’s Collaborative Center for Drug Statistics Methodology (Leucht et al. 2016). Then we used an independent sample t-test to assess the antipsychotic dose differences between the two groups. Statistical analysis showed that there was no significant difference in the mean dosage between the two groups (t(1,38) = 1.501, p = 0.142). We have added these clarifications in the Methods section.

References:

Leucht S, Samara M, Heres S, Davis JM. Dose Equivalents for Antipsychotic Drugs: The DDD Method. Schizophrenia Bulletin.2016;42(Suppl 1):S90-S94. doi:10.1093/schbul/sbv167.

[in Methods:]

‘All enrolled patients were treated with one or two second-generation oral antipsychotic drugs (e.g. olanzapine, risperidone, aripiprazole, clozapine, quetiapine, amisulpride). We chose olanzapine as the standard drug, for every patient, the cumulative antipsychotic dose was transformed into the equivalent dose to olanzapine based on defined daily doses (DDDs) presented by the WHO Collaborative Center for Drug Statistics Methodology [73]. Statistical analysis showed that there was no significant difference in the mean dosage between AVHs and non-AVHs groups (t(1,38) = 1.501, p = 0.142).’

*5) The definition of the non-AVH group needs to be clarified - is it a state or trait level - if trait level, the authors may wish to clarify how they ascertained an absolute lack of hallucinations throughout the illness.*

Re: We thank you for raising this constructive comment. In this study, the non-AVH group is defined at the trait level -- the non-AVH patients have never experienced AVH throughout the course of their illness. Evaluation criteria were based on 1) Comprehensive Clinical Interviews: Detailed interviews with patients, covering their entire psychiatric history, to confirm the absence of AVH. 2) Patient’s subjective report: By clearly defining what constitutes an AVH and making sure that patients understand the symptoms of AVH, and report that they have never experienced AVH. 3) Medical Records Review: Examination of medical records over the course of the illness to ensure no documented episodes of AVH. 4) Collateral Information: Gathering information from family members or caregivers who have been consistently involved in the patient's care to corroborate the absence of AVH. These strict criteria were used to better control and contrast with the AVH group to test our hypothesis. We have added these criteria in the methods section.

[in Methods:]

‘In this study, the non-AVHs group is defined at the trait level ― the non-AVHs patients have never experienced AVHs throughout the course of their illness. Evaluation criteria were based on 1) Comprehensive Clinical Interviews: Detailed interviews with patients, covering their entire psychiatric history, to confirm the absence of AVHs. 2) Patient’s subjective report: By clearly defining what constitutes an AVHs and making sure that patients understand the symptoms of AVHs, and report that they have never experienced AVHs. 3) Medical Records Review: Examination of medical records over the course of the illness to ensure no documented episodes of AVHs. 4) Collateral Information: Gathering information from family members or caregivers who have been consistently involved in the patient's care to corroborate the absence of AVHs. These strict criteria were used to better control and contrast with the AVHs group to test our hypothesis.’

Reviewer #2:

*This study explores how patients with schizophrenia distinguish reality from hallucinations, focusing on the roles of efference copy (EC) and corollary discharge (CD) in agency monitoring. Through EEG experiments involving patients with schizophrenia with and without auditory verbal hallucinations (AVHs), the authors find that general speech preparation does not suppress auditory responses due to CD dysfunction, while specific preparation improperly enhances auditory responses in AVH patients, indicating EC dysfunction. A computational model supports these findings, suggesting that the combination of impaired CD and EC functions leads to auditory hallucinations by causing erroneous internal auditory representation. This highlights the importance of motor-to-sensory transformation impairments in the positive symptoms of schizophrenia.*

*There are several issues with this study, in particular relating to the methodological approach taken, which makes it difficult to interpret the results.*

We thank you for perusing our manuscript and providing detailed suggestions. We appreciate your compliment on the novelty and significance of our study. According to the constructive suggestions, we have added experimental and methodological details. Moreover, we have performed a bootstrap simulation analysis and symptom-neural measure correlation analysis to address the potential issue of null results and sample size. We believe that the extensive clarifications, additional analyses and new results provide evidence to significantly boost the conclusiveness of the study, especially on the most crucial one about the null results. Please see our responses to each comment below.

*1) Analysis method: The computational model description is incomplete and it is not clear from the manuscript how the model was validated. How was the bifurcation analysis conducted? Was the model fit to the data, if so please plot the model fits and provide an assessment of the model accuracy.*

Re: We are sorry that we did not clearly present the model in the previous version of the manuscript. We built this processing model with a twofold purpose: 1) to quantitatively simulate the proposed impairment of distinct functional signals in motor-to-sensory transformation in auditory hallucinations, and 2) to frame the mechanisms in a biological-possible context so that future neuroscience studies can be better inspired. Therefore, we constructed a dedicated neural network simulating the interaction among the motor and auditory systems during speech production. The bifurcation refers to the model architecture that the motor signals split into two routes and provide distinct modulatory effects on the auditory system (in the new figure 6A in the manuscript, copied below as Fig. R2). Specifically, in one route, motor signals reach to all auditory neurons via an interneuron. The relay via an inhibitory interneuron induces suppression in the auditory processes (simulating the inhibitory function of CD); Whereas, in another direct route, motor signals directly exert influence on the corresponding auditory neurons for specifically increasing the gain of that auditory unit (simulating the enhancement function of EC).

Figure R2. **Model architecture.** Speech units in the motor layer (upper) link to the units in the auditory layer (lower) via two pathways. An interneuron receives signals from each unit in the motor layer and inhibits all units in the auditory layer (blue, simulating the inhibitory function of CD during the general preparation condition). Moreover, signals from each motor unit bifurcate and sensitize its corresponding auditory unit (red arrows, simulating the enhancement function of EC during the specific preparation condition). Using a scenario of preparing the syllable /ba/ as an example, the solid red arrow indicates the modulation in the corresponding prepared syllable, whereas the dashed arrows depict the lack of modulation in unprepared syllables.

The model is a simple rate model with built-in neural dynamics. The model was validated by fitting the simulated dynamic responses from the auditory output to the recorded EEG data. The fitting was at the level of overall measures ― temporal average of the simulated neural dynamic responses (just like obtaining the ERP components) and compared to the modulation effects in empirical auditory ERP responses (in the new Fig. 6B-F, and copied below as Fig. R3). Because this analysis was to test the null hypothesis that the model simulation results were from a similar distribution of empirical results, we used one-sample *t*-tests to test whether the mean estimated from the samples was different from the simulation results. Bayes factor analysis followed to provide the likelihood for the null hypothesis. The model simulation results were similar to the empirical results, quantified by one-sample tests with Bayes factor analysis ― the model performance cannot be distinguished from the distribution estimated by the empirical samples. We have elucidated the details of the model and fitting in the Methods section and copied below. Moreover, we have provided clarifications, especially about the hypothesized mechanisms (please also see our responses to the second question below).

Figure R3. **Simulation and model fitting results.** The simulation results of time course responses in B) GP and in C) SP conditions for normal (left), non-AVHs (middle), and AVHs (right) groups. Each line represents the simulated dynamics of responses from the corresponding auditory units, with black line for B condition, blue line for GP condition; solid and dashed red lines for SPcon and SPinc, respectively. The simulation results of component response magnitude compared with the empirical data in D) GP, E) SPcon, and F) SPinc conditions. The bars represent the observed modulation effects of speech preparation on N1 auditory responses. The stars on the bars represent the simulation results in a given condition and group. Both empirical and simulation results are normalized by baseline conditions. The model simulations capture the absence of inhibition during GP as well as the opposite modulation patterns in SP among different groups.

[in Methods:]

‘To assess how the model fitted the empirical results, the model was validated by fitting the simulated dynamic responses from the auditory output to the empirical EEG data. The fitting was at the level of overall measures – temporal averages of the simulated neural dynamic responses (analogs to the procedure of obtaining the ERP components) and compared to the modulation effects in auditory ERP responses. We treated the model simulation results as the mean from a distribution with unknown variance. Empirical ERP N1 component data in GP and SP conditions were subject to one-sample t-tests against the temporal averaged simulation results, separately for non-AVHs and AVHs groups. Because this analysis was to test the null hypothesis that the model simulation results were from a similar distribution of empirical results, we used a Bayesian analysis method for one-sample t-tests [74] (online tool at http://rdrr.io/rforge/BayesFactor/man/ttest.tstat.html). The Bayes factor is B01 = M0/M1, where M0 and M1 are the marginal likelihood for the null and alternative, respectively. That is, the Bayes factor is an odds ratio between the null and alternative hypotheses, which indicates that the null is B01 times more probable than the alternative. The parameters for the Bayesian analysis were a sample size of 20 and 20 for AVHs and non-AVHs groups and a scale r on an effect size of 0.707.’

*2) Clarity and specificity of the hypotheses: The study proposes intriguing hypotheses about the differential roles of corollary discharge and efference copy in schizophrenia. However, it could benefit from more precise definitions of "broken" CD and "noisy" EC, in particular in the context of the computational model used. The schematic figure and description is insufficient as it does not relate directly to the model used.*

Re: We thank you for precisely identifying and appreciating the novelty of the study. Using common words such as ‘broken’ and ‘noisy’, we intended to provide intuitive understanding for the general audience. We agree with the reviewer that it is beneficial to provide more precise definitions so that it is more operational to offer mechanistic insights and to inspire future studies. ‘Broken’ CD, equivalent to impaired CD, indicates the stop-working or close-to-stop working of the inhibitory function in patients, contrasting with the normal function that suppresses the auditory processes and arguably mediates the feeling of agency in the normal population. This impaired CD is indicated in the computational model as the lesion of the inter-neuron that carries the inhibitory function.

While ‘noisy’ EC is operationally defined as the unnecessary spread of activation to the motor neurons that are the neighbors of the articulatory target during specific speech preparation. The ‘noisy’ or broader activation of motor neurons contrasts with the precise activation of the articulatory target in the normal population where only the motor unit of the prepared syllable was activated. The unprecise activity in neighboring motor units sends signals directly to and increases the gain of the corresponding non-target auditory units, and hence boosts the erroneous activation of the auditory neurons. The unprecise motor activity and the downstream erroneous modulation on non-target (unprepared) auditory neurons constitute the ‘noisy’ EC. We have added these clarifications in the Introduction section, and updated the schematic model figure to more directly relate to the descriptions of the mechanistic definitions (the new Figure 6A in the manuscript, and copied below as Fig. R4).

Figure R4. **Model architecture and manipulations that quantify the hypothesized neural impairments mediating AVH**. Speech units in the motor layer (upper) link to the units in the auditory layer (lower) via two pathways. An interneuron receives signals from each unit in the motor layer and inhibits all units in the auditory layer (blue, simulating the inhibitory function of CD during general preparation). Moreover, signals from each motor unit bifurcate and sensitize its corresponding auditory unit (red arrows, simulating the enhancement function of EC during specific preparation). A trial that /da/ is prepared to speak is taken as an example, indicated by the only red in the motor ‘da’ neuron with a solid arrow pointing to the auditory ‘da’ neuron with a red circle. Other unprepared syllables do not activate the motor neurons and dashed arrows indicate no modulation on the corresponding auditory neurons. Compared with the normal population (left plot), for non-AVHs (middle plot), the weaker CD function is modeled as the reduced inhibition strength of the interneuron (indicated by a lightened red cross over the interneuron, with dashed blue lines representing reduced inhibition in downstream). Whereas the precision of the modulation strength of the EC enhancement function is preserved in non-AVHs, indicated by the same activation in the motor layer and modulation patterns to auditory neurons as the normal population (left). For AVHs (right plot), the inhibitory function of CD could be even more impaired (a red cross over the interneuron) and the enhancement function of EC is imprecise as activation in the motor layer spreads to other unprepared syllables and its modulation not only on the target unit of ‘da’ but also over all neighboring units (red in all motor units and solid arrows to all auditory units).

[in Introduction:]

‘whereas in patients with AVHs, in addition to the negative symptoms, the deficit in the self-monitoring of agency is manifested in the impairment of the inhibitory function of CD throughout the entire course of action – a ‘broken’ CD (Fig. 1C, the entire CD arrow is grey). When EC is available after specific movement plans have been formed, the enhancement function of EC on the prepared speech target is intact in non-AVHs patients (Fig. 1B, red arrow). Whereas, in patients with AVHs, the EC enhancement function is imprecise (Fig. 1C, hatched red arrow) and modulates both target reafference and its neighboring auditory units – a ‘noisy’ EC (potential causes of various perceptual-like auditory and verbal contents during hallucinations). That is, the positive symptoms of auditory hallucinations are mediated by the combination of a ‘broken’ CD and ‘noisy’ EC.’

*3) Sample size: The small sample sizes used in this study makes it difficult to assess the generalizability of the results, in particular the null effects. Can you reproduce these findings in a new sample?*

Re: We completely agree with the reviewer about the generalizability and replicability, especially for a novel study like the current one with a hypothesis that predicts null effects. Considering the costs and efforts that were determined by the uniqueness of the study ― cognitive tasks in clinical populations and strict control and inclusive criteria such as clearly defining the subgroups at trait level (it took us 5 years to recruit AVH and non-AVH patients and collect the data), we take a proactive approach to provide additional evidence and increase the conclusiveness of this study. Specifically, four independent aspects have been evaluated, including 1) a bootstrap resampling simulation to provide the confident interval for the null results; 2) correlations between symptom-neural measures that are complementary to the predicted null results; 3) multiple positive effects observed in the same sample, complementary to the predicted null results; and 4) ongoing follow-up studies serving the purpose of independent replication. Please see below for details.

1) Bootstrap analysis of resampling and estimating confidence interval

Due to the costs and efforts to conduct a direct replication of the observed null results in the GP condition, we implemented a bootstrapping procedure to estimate the reliability of the null results. Because the empirical sample of 20 participants was randomly drawn from the population, it should be representative of the population. Therefore, by resampling from the empirical sample many times with replacement, it would be as if many samples were drawn from the original population, and the estimates derived would be representative of the theoretical distribution (Chernick, 2011; Hall, 2013).

Specifically, we carried out the bootstrap analysis on the N1 component data of the GP condition in the AVH group where we predicted a null result derived from the ‘broken CD’ hypothesis. We first subtracted the baseline responses from the responses of the GP condition, yielding the GP modulation index for each individual. Then, we performed 10,000 times resampling with replacement and generated the estimated confidence interval. As the simulation results below, the suppression (modulation index value less than 0) is outside the 99% confidence interval, suggesting that the observed null results in the AVH group are highly unlikely due to chance. We have added these bootstrap results in supplementary materials as Fig. S2 and copied below as Fig. R5.

References:

Hall, P. (2013). The bootstrap and Edgeworth expansion: Springer Science &Business Media.

Chernick, M. R. (2011). Bootstrap methods: A guide for practitioners and researchers: John Wiley & Sons.

Figure R5. **Bootstrapping results of N1 component in GP condition of AVHs group**. The simulation is done by resampling with replacement 10,000 times in the GP modulation index of the empirical sample (GP minus baseline). The blue vertical line denotes the mean of the empirical sample. The horizontal red bar indicates the 99% confidence interval (CI). The suppression (modulation index value less than 0) is outside the CI.

2) Correlations between symptom severity and neural responses

According to our hypothesis that the CD function is impaired in the AVH group, the natural derivation of the hypothesis is that the severity of the hallucination symptoms correlates with the degree of impairment in the CD function. That is, in terms of neural measures, the severity of the symptoms would correlate with the measures of suppression in the GP condition. Therefore, we carried out Pearson correlation analysis between the GP modulation index (GP minus baseline) and three symptom scores (PTotal: positive symptoms score; NTotal: negative symptoms score; and AHRSTotal: auditory hallucination symptoms score). A significant positive correlation was observed between the PTotal (positive symptom score) and GP modulation index (after multiple-comparison correction) (included in the manuscript as the new Fig. 5A and copied below as Fig. R6). These significant results, contrasting with the original predicted negative results in the GP condition, provide additional strong evidence supporting the ‘broken CD’ hypothesis in schizophrenia patients.

Figure R6. **Significant correlation between the PTotal (positive symptom score) and GP modulation index (GP minus baseline)**

3) Positive results in other conditions from the same sample suggest enough power

In this study, we implemented a within-subject design where both GP and SP conditions were included in each of the AVH and non-AVH groups. While the suppression in the GP condition was not significant as predicted, the effects in the SP condition were significant in both groups. Together with the newly observed significant symptom-neural correlations, these positive results from the same sample suggest that we had enough power.

4) Ongoing projects using complementary methods and paradigms indicate the replicability of the results in the study.

Based on the proposed hypothesis and results of the current study, we have been conducting and will carry out several follow-up studies using fMRI, MEG, and neural modulations with related and extended paradigms to further investigate the proposed hypothesis regarding the motor-sensory relation with auditory hallucinations. The purpose of these follow-up studies is not only to replicate the observed results in the current study, but also to test additional aspects of the hypothesis. The preliminary results from these studies are promising and consistent with the proposed hypothesis. For example, we are using fMRI to investigate the inhibition control of overt and covert speech across normal, AVH and non-AVH groups. If CD has an inhibitory function for labeling the source that induces the neural representations, the AVH group would have deficits in the inhibition control network especially during covert speech (imagined speaking) when the control is purely in the mental space.

The preliminary results (Fig. R7 below, currently 20 participants in each group) show that in the condition that requires a stop of overt speech, comparing with normal and non-AVH groups, AVH group has significantly reduced activation in the inferior frontal cortex (IFC) where the initiation of inhibitory control has demonstrated; Whereas, similar activation is observed over temporal-parietal junction (TPJ) in all groups, consistent with the successful stop rate of overt speaking, suggesting the realization of inhibition control for physical action. (AVH has less IFC activation but similar responses in TPJ, probably because of relatively weaker initiation of CD for inhibition control and is compensated by other processes so that the inhibition of overt action can be achieved but with slower stopping reaction time.) During the condition that requires a stop of covert speech, a similar frontal-parietal neural network is observed for inhibition control of imagined speaking in all groups. More importantly, although similar activation in IFC in AVH groups suggests the efforts of initiation of inhibition control, the TPJ is absent compared with normal and non-AVH groups. These neural results are consistent with a less successful stop rate of covert speech in the AVH group (self-reported), suggesting the impairment in realizing the inhibition of mental activity. These results from ongoing fMRI projects are consistent with the hypothesis of ‘broken’ CD and ‘noisy’ EC and provide additional information probing the neural network that potentially mediates the control of motor-sensory interaction and auditory hallucinations.

Figure R7. Preliminary fMRI results of double-dissociation in the frontal-partial control network during a stop of overt and covert speech tasks in normal, non-AVHs and AVHs groups (currently 20 participants in each group). During the stop of overt speech, AVHs have significantly reduced activation in the inferior frontal cortex (IFC), while activity in the temporal-parietal junction (TPJ) is similar to the other two groups. During the stop of covert speech (imagined speaking), a similar frontal-parietal neural network is observed for inhibition control of imagined speaking in all groups. More importantly, although similar activation in IFC in AVH groups suggests the efforts of initiation of inhibition control, the TPJ is absent compared with normal and non-AVH groups. These results from ongoing fMRI projects are consistent with the proposed hypothesis of impaired motor-to-sensory transformation in AVH and provide additional information probing the neural network that potentially mediates the control of motor-sensory interaction and auditory hallucinations.

The extensive additional analyses and new results provide evidence to significantly boost the conclusiveness of the study, especially on the most crucial one about the null results. Moreover, the multiple follow-up experiments would fulfill the purpose of a direct replication, as well as testing new aspects of the hypothesis. This collaborative approach would be an optimal solution, after considering the costs and efforts of a direct replication, to demonstrate the validity of the project. Considering all the above, we believe that the results of the project are reliable -- letting the research community see the novel study would be beneficial and potentially inspire a new direction of research.

*4) EEG preprocessing and methodological details:* *how did the authors address ocular artifacts, what thresholds were used.*

Re: During EEG recording, we attached two electrodes (EOG) near the eyes to monitor the vertical and horizontal eye muscle movement. In EEG preprocessing, we utilized the 2 EOG and 32 EEG channels and created epochs based on EOG events (eye blinks or movements) and EEG events in the dataset. Initially, epochs containing conspicuous artifacts due to eye blinks or movement (generally > 100 µv) were manually excluded. Subsequently, we employed the Autoreject toolbox, a Python package designed for automated artifact rejection. This tool explicitly marks the EOG channels and correlates the timing of activation in the EOG channels with the EEG channels, and calculates an optimal rejection threshold for each channel using the ‘get_rejection_threshold’ function. This function assesses the signal characteristics of individual channels to determine appropriate peak-to-peak amplitude thresholds. Epochs exceeding these calculated thresholds were automatically rejected, ensuring our analysis was used the data that were free of significant ocular artifacts. We have added these methodological details in the Methods section.

[in Methods:]

‘During EEG recording, we attached two electrodes (EOG) near the eyes to monitor the vertical and horizontal eye muscle movement. In EEG preprocessing, we utilized the 2 EOG and 32 EEG channels and created epochs based on EOG events (eye blinks or movements) and EEG events in the dataset. Initially, epochs containing conspicuous artifacts due to eye blinks or movement (generally > 100 µv) were manually excluded. Subsequently, we employed the Autoreject toolbox, a Python package designed for automated artifact rejection. This tool explicitly marks the EOG channels and correlates the timing of activation in the EOG channels with the EEG channels, and calculates an optimal rejection threshold for each channel using the ‘get_rejection_threshold’ function. This function assesses the signal characteristics of individual channels to determine appropriate peak-to-peak amplitude thresholds. Epochs exceeding these calculated thresholds were automatically rejected, ensuring our analysis was confined to data free of significant ocular artifacts.’

*5)* *Comparison with existing literature: A more thorough comparison with existing literature on neurocomputational models of auditory hallucinations in schizophrenia would have placed the findings in a larger context and improved the readability of the discussion.*

Re: We thank you for this excellent suggestion. We have surveyed the major computational modeling of auditory hallucinations in schizophrenia published in the past fifteen years and include several representative ones in the discussion. Most of the previous modeling work focuses on the Bayesian inference framework by emphasizing the disfunction of prior in the induction of positive symptoms. In this study and model, we provide a biological possible mechanism that is consistent with the general Bayesian inference framework. Moreover, we emphasize the sensorimotor system and its computation as distinct functions to provide a neural explanation of generation and monitoring deficits in auditory hallucinations. The biological possible and novel sensorimotor framework could inspire further empirical studies from a neuroscience perspective at various levels. It is by no means an exclusive list, and we appreciate the reviewer as an expert on modeling to suggest more relevant papers if we missed important ones. We have added a paragraph of comparison with existing models in the Discussion section and copied below.

[in Discussion:]

‘Our neural network model aligns with previous Bayesian inference models that explain the positive symptoms of schizophrenia. False prediction errors have been assumed as the cause of the positive symptoms (Fletcher & Frith, 2009; Sterzer et al., 2018; Waltz et al., 2007). The inaccurate prior expectancies distort the integration with the sensory evidence, and the resultant false prediction errors create disturbance in inference updating. Specific to hallucinations, recent models propose that abnormally strong priors dominate and overwrite prediction errors so that both contents and the presence of auditory hallucinations can be explained (Corlett et al., 2019). Our model, based on two distinct modulatory functions in the motor-to-sensory transformation, assumes two types of predictions that can presumably integrate both inference models. EC serves as a content prediction, resembling the strong priors that would elicit the specific auditory neural representation without corresponding external stimulation (Chu et al., 2023; Li et al., 2020; Tian et al., 2018; Tian et al., 2016). Whereas, CD serves as a prediction for agency, and the impairment would cause the failure to explain away the internally generated auditory neural representations that are evoked by EC (Ma & Tian, 2019; Yang et al., 2019; Yang et al., 2021; Zheng et al., 2022). Moreover, our neural network model provides a mechanistic account and potentially ground the psychological constructs of inference and prediction in the motor and sensory systems. The hypothesized origins of auditory hallucinations at the level of neural representation and computation can inspire future neuroscience studies at the cellular, system, and cognitive levels. ’

References:

Chu, Q., Ma, O., Hang, Y., & Tian, X. (2023). Dual-stream cortical pathways mediate sensory prediction. *Cerebral Cortex, 33*(14), 8890-8903. doi:10.1093/cercor/bhad168

Corlett, P. R., Horga, G., Fletcher, P. C., Alderson-Day, B., Schmack, K., & Powers, A. R. (2019). Hallucinations and strong priors. *Trends in Cognitive Sciences, 23*(2), 114-127.

Fletcher, P. C., & Frith, C. D. (2009). Perceiving is believing: a Bayesian approach to explaining the positive symptoms of schizophrenia. *Nature Reviews Neuroscience, 10*(1), 48-58.

Li, Y., Luo, H., & Tian, X. (2020). Mental operations in rhythm: Motor-to-sensory transformation mediates imagined singing. *PLoS Biology, 18*(10), e3000504.

Ma, O., & Tian, X. (2019). Distinct mechanisms of imagery differentially influence speech perception. *eneuro, 6*(5).

Sterzer, P., Adams, R. A., Fletcher, P., Frith, C., Lawrie, S. M., Muckli, L., . . . Corlett, P. R. (2018). The predictive coding account of psychosis. *Biological Psychiatry, 84*(9), 634-643.

Tian, X., Ding, N., Teng, X., Bai, F., & Poeppel, D. (2018). Imagined speech influences perceived loudness of sound. *Nature Human Behaviour, 2*(3), 225-234.

Tian, X., Zarate, J. M., & Poeppel, D. (2016). Mental imagery of speech implicates two mechanisms of perceptual reactivation. *Cortex, 77*, 1-12.

Waltz, J. A., Frank, M. J., Robinson, B. M., & Gold, J. M. (2007). Selective reinforcement learning deficits in schizophrenia support predictions from computational models of striatal-cortical dysfunction. *Biological Psychiatry, 62*(7), 756-764.

Yang, F., Fang, X., Tang, W., Hui, L., Chen, Y., Zhang, C., & Tian, X. (2019). Effects and potential mechanisms of transcranial direct current stimulation (tDCS) on auditory hallucinations: A meta-analysis. *Psychiatry Research, 273*, 343-349.

Yang, F., Zhu, H., Yu, L., Lu, W., Zhang, C., & Tian, X. (2021). Deficits in multi-scale top-down processes distorting auditory perception in schizophrenia. *Behavioural Brain Research, 412*, 113411.

Zheng, X., Zhu, H., Li, S., & Tian, X. (2022). The generic inhibitory function of corollary discharge in motor intention: evidence from the modulation effects of speech preparation on the late components of auditory neural responses. *eneuro, 9*(6).
